# Overcoming Resistance in Anderson–Fabry Disease: Current Therapeutic Challenges and Future Perspectives

**DOI:** 10.3390/jcm13237195

**Published:** 2024-11-27

**Authors:** Maria Cristina Carella, Cinzia Forleo, Pierpaolo Caretto, Maria Ludovica Naccarati, Ilaria Dentamaro, Marco Maria Dicorato, Paolo Basile, Eugenio Carulli, Michele Davide Latorre, Andrea Baggiano, Gianluca Pontone, Marco Matteo Ciccone, Andrea Igoren Guaricci

**Affiliations:** 1 Interdisciplinary Department of Medicine, University of Bari “Aldo Moro”, Polyclinic University Hospital, 70124 Bari, Italy; m.carella31@phd.uniba.it (M.C.C.); cinzia.forleo@uniba.it (C.F.); p.caretto@studenti.uniba.it (P.C.); marialudovica97@libero.it (M.L.N.); ilaria.dentamaro@policlinico.ba.it (I.D.); m.dicorato20@studenti.uniba.it (M.M.D.); paolo.basile@uniba.it (P.B.); micheledavide.latorre@policlinico.ba.it (M.D.L.); marcomatteo.ciccone@uniba.it (M.M.C.); 2 Department of Emergency and Acceptance, Division of Cardiology, Azienda Sanitaria locale Matera, 75100 Matera, Italy; e.carulli@phd.uniba.it; 3 Department of Perioperative Cardiology and Cardiovascular Imaging, IRCCS Centro Cardiologico Monzino, 20138 Milan, Italy; andrea.baggiano@cardiologicomonzino.it (A.B.); gianluca.pontone@cardiologicomonzino.it (G.P.); 4 Department of Biomedical, Surgical and Dental Sciences, University of Milan, 20122 Milan, Italy

**Keywords:** Anderson–Fabry disease, enzyme replacement therapies, resistance mechanisms, chaperone therapy, mRNA, gene therapies, anti-drug antibodies, tailoring treatment

## Abstract

Anderson–Fabry disease (AFD) remains a therapeutic challenge despite advances in early diagnosis and the availability of enzyme replacement therapies (ERTs). While early initiation of therapy can mitigate disease progression, resistance mechanisms—such as the development of anti-drug antibodies—limit the efficacy of current treatments, particularly in patients with severe genetic variants. Chaperone therapy provides a targeted option for a subset of patients, yet significant gaps remain in treating those with complete enzyme deficiency. This perspective article explores the existing therapeutic landscape and reflects on emerging treatments, such as mRNA and gene therapies, which hold promise for overcoming the resistance mechanisms. By addressing the limitations of current pharmacological options and considering future innovations, this article aims to outline the path forward for more effective and personalized treatment strategies in Anderson–Fabry disease.

## 1. Introduction

The diagnostic pathways of all cardiomyopathies are currently characterized by considerable technological implementation, which has led to an increased evidence base in the general population [1,2,3,4,5,6,7]. This progress has highlighted the need to optimize therapeutic strategies, particularly for challenging pathologies like Anderson–Fabry disease (AFD), where treatment resistance and variability in clinical outcomes remain significant concerns [8,9,10].

AFD is an X-linked metabolic disorder caused by pathogenic variants in the *GLA* gene, leading to reduced or absent activity of α-galactosidase A [11,12,13,14]. This enzyme deficiency results in the lysosomal accumulation of globotriaosylceramide (Gb3) and its deacetylated form (LysoGb3) in various tissues, including the heart, kidneys, and nervous system [15,16]. The systemic nature of AFD often results in multi-organ involvement, with cardiac complications being a leading cause of morbidity and mortality. These complications include hypertrophic cardiomyopathy, arrhythmias (e.g., atrioventricular block, ventricular, and supraventricular tachycardia) [17], and progressive heart failure [18]. Cardiac manifestations are not only the primary contributors to adverse prognosis but they also significantly impair the quality of life for affected patients [19].

Despite increased attention to this disease, therapeutic outcomes remain suboptimal. Early diagnosis is critical, as delayed initiation of disease-specific therapy often leads to irreversible organ damage and limited therapeutic efficacy. However, even when treatment is initiated promptly, challenges persist, particularly due to the development of anti-drug antibodies against agalsidase alpha and beta, which are the key components of enzyme replacement therapy (ERT) [20]. ERT remains the primary therapeutic option for patients with genetic variants that result in complete enzyme deficiency, yet its efficacy is limited by immunogenic reactions. Moreover, the limited ability of ERT to fully reverse disease progression highlights the need for complementary or alternative therapeutic options. In recent years, chaperone therapy has emerged as an alternative for patients with amenable genetic variants, stabilizing the enzyme and preventing its degradation. However, this approach is limited to specific mutations and does not address the broader challenge of treatment resistance [21].

The emergence of novel therapeutic technologies such as gene therapy and mRNA-based treatments marks a promising paradigm shift in AFD management. Gene therapy, using both ex vivo and in vivo approaches, aims to correct the underlying enzymatic defect, potentially offering a curative solution [22,23]. Similarly, mRNA therapies hold promise in delivering functional α-galactosidase A directly to affected cells [22,23]. These advanced therapies have shown potential in preclinical and early clinical studies, with improvements in Gb3 clearance and reductions in systemic biomarkers such as Lyso-Gb3.

Despite advances in early diagnosis and available therapies, significant gaps remain in the management of AFD, particularly concerning therapeutic resistance. This perspective aims to provide a comprehensive overview of the mechanisms underlying therapy resistance and to explore how these innovative approaches could overcome current barriers, ultimately leading to improved outcomes for patients.

## 2. Mechanisms of Therapy Resistance

ERT with recombinant α-galactosidase A is currently the cornerstone of treatment for AFD in patients with *GLA* gene mutations resulting in a complete loss of enzyme function. Two formulations are available: agalsidase-α and agalsidase-β. While a recent review of randomized trials revealed no significant difference between the two in terms of renal damage, cardiac events, mortality, or reduction in Gb3 levels, in patients treated with agalsidase-α compared to agalsidase-β [24], the therapeutic landscape for AFD remains complex and far from optimal [20]. Despite its efficacy in reducing left ventricular mass index (LVMI) and left ventricular hypertrophy (LVH) when initiated early, ERT faces significant limitations due to a variety of resistance mechanisms that extend beyond the timing of therapy initiation. Overall, the poor biodistribution, the short half-life, and the immunogenic responses, especially in key organs like the heart, kidney, and brain, are the causes of therapy resistance [25,26]. Moreover, the high cost (~USD 200,000/year) and reduced efficacy in end-stage disease or in patients with antibodies also pose challenges [27].

The complexity of therapy resistance in AFD can be summarized in some key mechanisms (Table 1).

(a) **Incomplete Gb3 clearance:** Although the timely initiation of ERT is beneficial for cardiac manifestations, its effects can be limited, particularly in cardiomyocytes. Histological studies have shown that Gb3 clearance is more efficient in endothelial cells than in cardiomyocytes, leading to suboptimal outcomes in cardiac tissue [28].
(b) **Antibody formation:** A considerable proportion of patients, especially males with the classic form of AFD, develop anti-drug antibodies (ADAs) against the recombinant enzyme [29], neutralizing its activity and reducing its effectiveness [30]. ERT is composed of a foreign recombinant protein that has the potential to stimulate a humoral immune response. ADAs typically develop within 3-6 months of starting therapy [31,32] and remain permanent [33,34]. The likelihood of ADAs’ development seems to be higher in patients with a more severe, classic phenotype [35] and in those treated with agalsidase-β [36]. Furthermore, elevated baseline levels of Gb3 have been linked to an increased risk of ADA formation [37], which appears to affect the course and severity of the disease [34].
(c) **Genetic variability:** Different mutations in the *GLA* gene result in a range of residual enzyme activity, influencing both the severity of the disease and the response to ERT. Certain mutations lead to poor therapeutic outcomes [15]. Furthermore, sometimes it is possible that an “overlap syndrome” characterized by the presence of both sarcomeric and Anderson–Fabry-related genetic variants, presents additional challenges to effective treatment.
(d) **LVH severity:** Data suggest that ERT is more effective in stabilizing or reducing LVH particularly in patients with mild to moderate hypertrophy [38,39,40,41]. On the other hand, studies suggest that when therapy is initiated in patients with advanced LVH, the progression of hypertrophy and subsequent complications is often inevitable, despite treatment [42].
(e) **Fibrosis and irreversible damage:** Chronic Gb3 accumulation results in irreversible myocardial fibrosis, a condition that ERT cannot reverse. The benefits of early ERT initiation are evident in patients who have not yet developed significant fibrosis, as demonstrated in several studies [17,42,43,44,45]. It is unlikely that patients with evidence of CMR findings suggestive of fibrosis experience regression of LVH [46,47,48,49,50]. A meta-analysis of randomized and observational trials highlighted sex-based differences in response to ERT. In male patients with AFD and baseline LVH, ERT led to stabilization of the left ventricular mass index (LVMI). Conversely, in male patients without baseline LVH, those treated with ERT showed a slower increase in LVMI over time. Among female patients, ERT resulted in a reduction in LVMI in those with baseline LVH, and stabilization of LVMI in those without baseline LVH [51].
(f) **Suboptimal dosage and timing:** Delayed diagnosis and late initiation of therapy, as well as suboptimal dosing, contribute to the limited effectiveness of ERT. Early and adequate dosing are critical for preventing irreversible organ damage [15].
(g) **Drug metabolism variability:** Some patients under ERT show disease progression, typically with renal, cardiovascular, and cerebrovascular dysfunctions. The existing literature provides evidence that gene variants involved in drug absorption, distribution, metabolism, and excretion may contribute to the variability in therapeutic response. Although with a limited sample size, Scionti et al. identified a statistically significant correlation between specific polymorphisms in the gene that codes for alcohol dehydrogenase and the progression of cardiac and renal disease, pointing to the importance of pharmacogenetics in understanding therapy resistance [52].

## 3. Therapeutic Alternatives in Anderson–Fabry Disease: Future Directions and Challenges

The therapeutic armamentarium for Anderson–Fabry disease is limited, with only a few validated and recommended tools available, according to current guidelines. Despite the progress in developing therapies, responses remain inconsistent due to several factors that influence treatment outcomes. This raises important questions about how we can further optimize therapeutic strategies for AFD.

**Figure 1:**
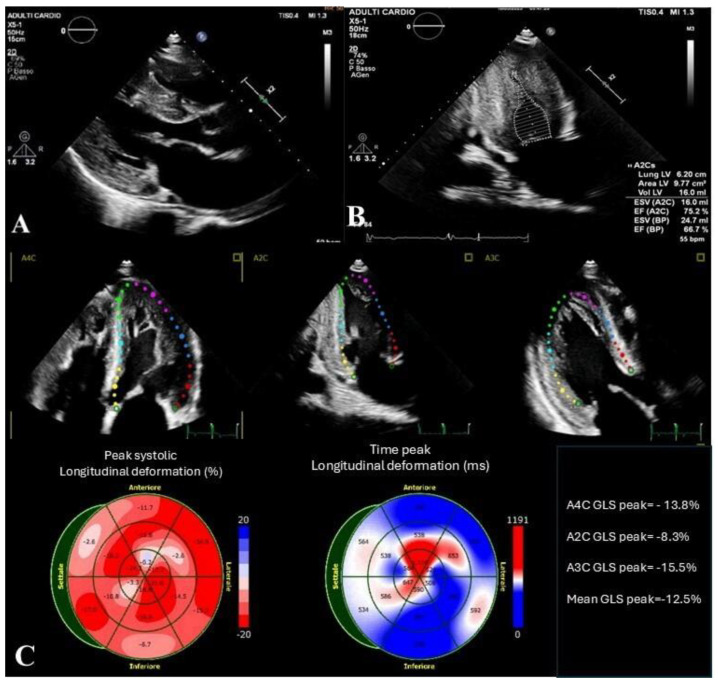
Case of a 57year-old male with Anderson–Fabry disease and coexisting sarcomeric mutations. The patient, initially presenting with mild left ventricular hypertrophy and recurrent palpitations, underwent extensive evaluation following the discovery of cornea verticillata and angiokeratomas. Genetic testing confirmed a pathogenic *GLA* gene variant, diagnostic of Anderson–Fabry disease, along with two sarcomeric variants of uncertain significance. Cardiac MRI revealed intramyocardial edema and late gadolinium enhancement (LGE), while blood tests showed elevated LysoGb3 levels. ERT with agalsidase-α was initiated, resulting in transient clinical improvement. However, the patient demonstrated progressive ventricular hypertrophy, increased cardiac mass, and a small cerebral hemorrhage on MRI. Persistent acroparesthesias and fluctuating LysoGb3 levels, despite ERT, indicated resistance to therapy. It is important to note that the sarcomeric mutations identified were of uncertain clinical significance and were unlikely to influence the AFD phenotype or the fluctuating LysoGb3 levels but may have contributed to the overall complexity of the cardiomyopathy phenotype observed. (**A**) Parasternal long-axis view shows severe concentric hypertrophy of the left ventricle; (**B**) left ventricular ejection fraction measured by the Simpson biplane method is preserved; (**C**) a reduction is detected in the GLS values.

One critical question is whether delayed diagnosis impacts prognosis, as early initiation of treatment is key to preventing irreversible organ damage. Another area of interest is the role of genetic variability, such as the presence of sarcomeric variants, which may exacerbate cardiomyopathy and complicate treatment (Figure 1). Additionally, understanding whether a patient develops one of the known resistance mechanisms, such as the formation of ADAs, is crucial for tailoring future therapeutic approaches.

Despite the limited number of available therapies, the variability in treatment response is vast (Table 2). Resistance mechanisms, including ADA formation, incomplete Gb3 clearance, and initiation of therapy at advanced stages when fibrosis is already present, represent significant hurdles.

However, novel therapeutic options are emerging to address these limitations. For instance, another available clinical approach for treating Anderson–Fabry disease is chaperone therapy, which stabilizes the *GLA* protein conformation in the endoplasmic reticulum, enhancing its proper folding and lysosomal trafficking. This mechanism prolongs the enzyme’s half-life and improves its stability [53]. Chaperone therapies are classified into three categories based on their interaction with the enzyme: exogenous competitive chaperones, which directly bind the active site of the enzyme to stabilize its conformation; exogenous non-competitive chaperones, which bind other regions of the enzyme, improving its overall stability; and endogenous molecular chaperones, which are naturally produced proteins aiding enzyme folding and stability [54]. Among these, migalastat, an orally available competitive chaperone, represents the most well-established agent for Anderson–Fabry therapy [55]. By selectively binding to and stabilizing mutant forms of α-Gal A with residual activity, migalastat enhances lysosomal enzyme levels and functionality [55]. Its oral administration offers a significant advantage over intravenous ERT, improving patient adherence and quality of life. Furthermore, its ability to cross the blood–brain barrier extends its therapeutic potential to neurological manifestations of the disease [56]. Clinical studies have demonstrated that migalastat increases α-Gal A activity and reduces disease biomarkers, such as serum creatinine and plasma globotriaosylsphingosine (Lyso-Gb3). A pivotal trial reported a 25% reduction in left ventricular mass index (LVMI) after one year of migalastat therapy, alongside stabilization of renal function and reductions in proteinuria [55]. However, the efficacy of migalastat is contingent upon the presence of residual enzyme activity [57] and amenable GLA mutations, which account for approximately 35–50% of all cases [21,58]. For this reason, an in vitro assay of responsiveness to treatment is recommended [59]. This leaves a subset of patients—those with non-amenable variants—relying solely on ERT, where the development of ADAs continues to be a major challenge.

Agalsidase-α and agalsidase-β, both recombinant forms of α-galactosidase A, have demonstrated efficacy in reducing Gb3 accumulation in plasma, urine, and tissues, with clinical benefits including stabilization of renal function and reduced left ventricular mass [15,60]. Agalsidase-α and agalsidase-β are two highly immunogenic recombinant proteins, which carry the risk of triggering an immune response against the patient’s own antigens. However, their immunogenicity remains a significant limitation, with ADAs developing in up to 88% of male patients [15,60]. To overcome this problem, studies are currently underway to assess the efficacy of alternative drugs with a similar mechanism of action to agalsidase-α and -β, but with significantly reduced immunogenicity: pegunigalsidase-α is a pegylated, covalently cross-linked form of agalsidase-α whose role in the treatment of AFD is being investigated in two phase III trials (NCT03180840 and NCT04552691). The pegylated, cross-linked structure has shown a significantly longer half-life and seems to reduce immunogenicity [61,62], offering a potential solution to the problem of developing ADAs. This drug could also be potentially administered in patients previously treated with agalsidase-α who have developed autoantibodies. In fact, ADAs seem to have a reduced affinity for pegunigalsidase-α [63]. This could represent a breakthrough in managing patients who have shown poor responses to standard ERT.

ERT remains a cornerstone of treatment, and efforts are underway to enhance its efficacy through pharmacological chaperones such as galactose [64]. Galactose has been shown to stabilize recombinant α-galactosidase A (rh-AGAL), improving its intracellular stability and enzymatic activity [64]. This co-administration approach could reduce the frequency or dosage of ERT while maintaining therapeutic benefits [64].

Moss-aGal represents an innovative advancement in enzyme replacement therapy, utilizing the moss Physcomitrella patens as a production host [23,65]. This novel platform challenges the established belief that mannose-6-phosphate modification is essential for lysosomal targeting, as moss-aGal has demonstrated effective cellular uptake via mannose receptors in preclinical studies [23,66]. Early-phase clinical trials confirmed its safety, with no significant adverse immune responses and reductions in Gb3 levels. Moss-aGal offers a unique alternative to traditional mammalian cell-derived enzymes, potentially improving therapeutic accessibility and cost-effectiveness while maintaining efficacy [23,67].

Recent studies have also identified α-synuclein (SNCA) as a substrate-independent contributor to Anderson–Fabry nephropathy [68]. SNCA accumulation leads to lysosomal dysfunction and oxidative stress, which persist despite ERT [68]. Preclinical models demonstrated that β2-adrenergic receptor agonists, such as clenbuterol and orciprenaline, can reduce SNCA levels and ameliorate lysosomal dysfunction, suggesting their potential as complementary therapies to ERT [68]. However, the pro-arrhythmogenic effects of β2-agonists pose a challenge for their use in Anderson–Fabry patients with cardiac involvement [68].

Nanocarrier-based strategies represent a cutting-edge approach to improving the delivery and efficacy of therapies for AFD. These systems are designed to enhance the bioavailability and cellular uptake of therapeutic agents such as *GLA*, particularly in target tissues like the vascular endothelium and myocardium. By optimizing the pharmacokinetics and biodistribution of *GLA*, nanocarriers offer a promising solution to some of the limitations associated with conventional ERT [15,23]. Arginine–glycine–aspartic acid (RGD)-coated liposomes are among the most advanced nanocarrier systems developed for AFD. These liposomes target integrins, which are highly expressed on endothelial cells, ensuring precise delivery of *GLA* to vascular tissues. This targeted approach not only improves bioavailability but also enhances colloidal stability, reducing the degradation of the therapeutic agent during systemic circulation [69]. However, challenges remain in overcoming biological barriers inside the cell. Lysosomal-targeting systems using pH-responsive or enzyme-targeted nanocarriers have shown promise in delivering *GLA* [70,71]. Although nanoplatforms have advanced *GLA* delivery, key obstacles include complexity in design, limited preclinical evaluation, and challenges in addressing CNS manifestations due to the blood–brain barrier [72]. Long-term biosafety and material toxicity also require further investigation for clinical translation.

High-throughput screening (HTS) has emerged as a powerful tool in the search for repositioned drugs targeting Anderson–Fabry disease and other lysosomal storage disorders [73]. This approach allows the rapid screening of extensive compound libraries for potential therapeutic effects. In Anderson–Fabry disease, HTS-guided drug repositioning has identified pharmacological chaperones and lysosomal function modulators [73]. For instance, acetylsalicylic acid and curcumin have shown potential as adjuncts to ERT, stabilizing AGAL and enhancing its long-term activity [73,74,75]. Similarly, HTS has identified compounds like deferoxamine and luteolin, which modulate lysosomal function and autophagic pathways, offering new avenues for therapeutic development [73,76,77].

As has been demonstrated in the case of Gaucher disease, a promising new avenue for the treatment of Anderson–Fabry disease is substrate reduction therapy (SRT). SRT involves using molecular drugs to reduce the storage of undigested macromolecules by inhibiting the synthesis of their substrates [78]. SRT has advantages such as high bioavailability and the ability to cross the blood–brain barrier, but it requires significant residual enzyme activity to be effective. In the context of Anderson–Fabry disease, drugs like Lucearstat and Venglustat have been developed to reduce Gb3 and LysoGb3 accumulation, particularly in tissues like the brain, which are difficult to reach with enzyme therapy [79,80,81]. Lucearstat, a glucosylceramide synthase inhibitor, has shown promising results in reducing plasma Gb3 and improving organ pathology in clinical trials (NCT02930655). However, phase three trials revealed it failed to alleviate neuropathic pain in Anderson–Fabry patients (NCT03425539). Venglustat, another SRT drug, also inhibits glucosylceramide synthase and has shown potential in phase two trials for Anderson–Fabry disease (NCT02228460), with ongoing studies assessing its long-term efficacy and safety (NCT02489344). Beyond their primary targets, these drugs also hold potential for combination therapies. For instance, pairing SRT with ERT or other emerging therapies could enhance overall efficacy by addressing multiple aspects of Anderson–Fabry disease pathophysiology [15,60]. SRT thus represents a significant step forward in expanding the therapeutic options for Anderson–Fabry disease, potentially addressing unmet needs for patients with central nervous system involvement and those with resistance or limited response to existing therapies [15,60]. Despite these advancements, challenges remain. Both Lucearstat and Venglustat need further evaluation in larger and more diverse patient populations to confirm their therapeutic benefits and safety profiles.

A further emerging approach is gene therapy. This can be categorized into two main approaches: viral vector-mediated gene therapy, which uses viruses to efficiently deliver and express therapeutic genes, and non-viral vector-mediated gene therapy, which utilizes alternatives such as small interfering RNA (siRNA), messenger RNA (mRNA), or plasmid DNA for gene delivery.

Non-viral methods offer reduced toxicity and immunogenicity compared with viral vectors. However, their low stability and transfection efficacy limit their effectiveness. mRNA therapies encode recombinant α-galactosidase and are currently in the experimental phase and undergoing testing in primates and rats. The target is represented by hepatocytes, where the α-galactosidase A enzyme is produced and secreted from mRNA inoculated with administered lipid vectors intravenously every six weeks [22,82]. The resulting enzyme is endogenous with glycosylation profiles that do not elicit an autoimmune response, and there is no risk of mutation linked to insertion defects [83]. This represents a potential game-changer for the long-term management of AFD.

In addition to mRNA therapy, viral vector-mediated gene therapy holds immense potential. By using lentivirus-mediated transduction of *GLA* DNA into autologous hematopoietic stem/progenitor cells, researchers have demonstrated sustained increases in α-galactosidase activity in a limited number of patients [84]. However, challenges remain, as evidenced by discordant results from the AVR-RD-01 program, which led to its early termination [23]. In vivo approaches utilize adeno-associated viral (AAV) vectors, such as 4D-310 and FLT190, to deliver the functional *GLA* gene directly into patients [23]. Initial data suggest that despite sustained α-Gal A activity and reductions in Gb3 levels, there were safety concerns, including mild myocarditis, which require further evaluation [23].

CRISPR/Cas9 gene editing represents a groundbreaking approach to addressing the root cause of AFD by directly targeting and correcting mutations in the *GLA* gene [50,85]. This strategy offers the potential for a permanent cure by restoring the endogenous production of functional α-galactosidase A, thereby eliminating the need for lifelong ERT or other adjunctive treatments [50,85]. The CRISPR/Cas9 system utilizes a guide RNA (gRNA) to direct the Cas9 nuclease to a specific target sequence within the *GLA* gene. Once the target site is identified, Cas9 introduces a double-strand break (DSB), which can then be repaired by one of two cellular pathways: non-homologous end joining (NHEJ), that often results in small insertions or deletions (indels) that may disrupt the defective gene sequence, and homology-directed repair (HDR), a more precise mechanism that uses a donor DNA template to correct the mutation, enabling the restoration of normal α-Gal A function [50,85]. Studies using in vitro and animal models have provided promising insights into the application of CRISPR/Cas9 for Anderson–Fabry disease. In vitro, the technology has been employed to correct *GLA* mutations in induced pluripotent stem cells (iPSCs) derived from Fabry patients [15,60]. Following the genetic correction, these edited cells exhibited restored α-galactosidase A activity, coupled with a significant reduction in Gb3 accumulation, a hallmark of Anderson–Fabry disease pathology [15,60]. In parallel, animal studies have demonstrated the feasibility of effectively delivering CRISPR/Cas9 constructs via AAV vectors. Preclinical experiments in mice revealed that this delivery approach successfully enhanced α-Gal A expression while reducing Gb3 deposition in key tissues affected by the disease, including the cardiac, renal, and vascular systems. These findings highlight the potential of CRISPR/Cas9 not only to target the genetic basis of Anderson–Fabry disease but also to ameliorate its systemic manifestations [15,60]. Despite its transformative potential, the application of CRISPR/Cas9 in Anderson–Fabry disease faces several critical challenges. Off-target effects remain a significant concern, as unintended edits in non-targeted genomic regions could lead to unpredictable consequences, including oncogenesis. Efficient and tissue-specific delivery mechanisms also present a hurdle, with current approaches like AAV vectors limited by their immunogenicity and payload capacity. Moreover, the reliance on HDR for precise gene correction is problematic, given its low activity in non-dividing cells such as cardiomyocytes and neurons, which are central to Anderson–Fabry disease. Ethical and regulatory concerns further complicate its implementation, as the long-term safety and potential consequences of genome editing, particularly in germline cells, require careful oversight and evaluation. To address these limitations, ongoing research is exploring innovative strategies. Next-generation CRISPR systems, including base editors and prime editing, aim to achieve precise single-nucleotide corrections without introducing double-strand breaks, thereby reducing the risk of off-target effects [85,86]. Non-viral delivery methods, such as lipid nanoparticles, are being developed to enhance safety and tissue specificity while avoiding the limitations of viral vectors [85,86]. Advances in multiplex editing with multi-guide RNA strategies also show promise in correcting multiple pathogenic variants simultaneously, addressing the genetic heterogeneity of Anderson–Fabry disease [85,86]. Although still in its infancy, CRISPR/Cas9 gene editing holds tremendous promise for revolutionizing Anderson–Fabry disease treatment by directly targeting the underlying genetic defect. This approach has the potential to achieve long-term remission or even a cure, eliminating the need for lifelong therapies. However, rigorous preclinical and clinical testing will be critical to ensure its safety, efficacy, and practical feasibility. If these barriers are overcome, CRISPR/Cas9 could herald a paradigm shift in managing Anderson–Fabry disease, offering patients a future with transformative therapeutic possibilities.

These emerging therapies offer new hope for overcoming the limitations of current treatments. However, many of these modalities are still in the preclinical or early clinical trial stages, and their efficacy and safety must be rigorously evaluated before they can be integrated into clinical practice. Nevertheless, the advent of mRNA therapy, gene therapy, and SRT represents a significant leap forward, bringing us closer to a future where personalized medicine can offer long-term solutions for patients with Anderson–Fabry disease. While challenges remain, these innovations signal a new era of treatment for Anderson–Fabry disease, with the potential to not only address therapeutic resistance but to also transform the prognosis for affected individuals.

## 4. Conclusions

Cardiac involvement in Anderson–Fabry disease significantly impacts patient outcomes and presents challenges in treatment, primarily due to therapy resistance mechanisms. Tailoring treatment based on individual genetic and biochemical profiles may improve outcomes. Early and accurate diagnosis, along with continuous monitoring, are crucial components of this approach. As our understanding of the disease’s underlying mechanisms deepens, so does the potential for novel therapeutic strategies to reshape the treatment landscape. The emergence of pegylated agalsidase-α, gene therapy, and mRNA-based treatments heralds a new era in the management of Anderson–Fabry disease. These innovative approaches offer the potential to overcome existing resistance mechanisms, but their long-term efficacy and safety remain to be confirmed through robust clinical trials.

Looking ahead, the integration of these novel therapies into clinical practice could revolutionize the management of Anderson–Fabry disease, moving us toward a more tailored and effective approach. While challenges persist, the future of treatment lies in the ability to personalize therapies and harness emerging technologies to mitigate the impact of therapeutic resistance, ultimately improving both survival and quality of life for patients with Anderson–Fabry disease.

## Figures and Tables

**Table 1:** Mechanisms of therapy resistance in Anderson–Fabry disease. α-*GLA*: α-galactosidase A; Gb3: globotriaosylceramide; ERT: enzyme replacement therapy; LVH: left ventricular hypertrophy.

Therapy Resistance Mechanism	Impact on ERT Effectiveness	Proposed Solutions
**Incomplete Gb3ΔClearance**	Reduces overall therapeutic benefits due to residual substrate.	Earlier intervention and adjunctive therapies.
**Antibody Formation**	Antibodies neutralize drug, reducing effectiveness.	Immunomodulation or alternative ERT formulations.
**Genetic Variability**	Different α-*GLA* mutations affect disease severity and ERT response. Possible “overlapping” between different pathogenic genetic variants.	Personalized approaches based on genetic profiles.
**LVH Severity**	More effective in mild/moderate LVH; less in advanced cases.	Early diagnosis and initiation of therapy.
**Fibrosis**	Advanced fibrosis limits benefits; early ERT is better.	Combination therapies targeting fibrosis pathways.
**Dosage and Timing**	Delayed or suboptimal dosing reduces effectiveness.	Optimized dosing strategies based on clinical needs.
**Drug Metabolism**	Polymorphisms of genes coding for enzymes involved in drug metabolism could alter drug response.	Genotyping to tailor ERT regimens.

**Table 2:** Available and investigational treatments for Anderson–Fabry disease (AFD). α-*GLA*: α-galactosidase A; mRNA: messenger RNA.

Drug/Therapy	Mechanism of Action	Indication	Recommendations
Agalsidase-α	Enzyme replacement therapy (ERT). Replacing of enzyme deficiency.	Classic and non-classic Anderson–Fabry disease.	AFD with complete α-*GLA* loss of function.
Agalsidase-β	Enzyme replacement therapy (ERT). Replacing of enzyme deficiency.	Classic and non-classic Anderson–Fabry disease.	Regular infusions, typically higher dose than agalsidase-α in AFD with complete α-*GLA* loss of function.
Migalastat	Pharmacological chaperone; enzyme’s stabilization and transport in the lysosome.	Specific amenable *GLA* mutations.	Oral administration, for patients with amenable mutations only.
Substrate Reduction Therapy (SRT)	Antagonism of glucosylceramide synthase and reduction in substrate accumulation.	Early intervention in Anderson–Fabry disease.	Still under clinical investigation. (Lucearstat: NCT02930655– NCT03425539; Venglustat: NCT02228460–NCT02489344).
Gene Therapy	Gene replacement; delivery of a functional α-*GLA* gene to patients.	Experimental for Anderson–Fabry disease.	Ongoing clinical trials (NCT02800070–NCT04040049).
mRNA Therapy	mRNA inoculation: use of mRNA to produce α-*GLA* enzyme in cells.	Experimental for Anderson–Fabry disease.	Evaluated in preclinical studies.

## Data Availability

Data sharing is not applicable.
